# High *In Situ* Repeatability of Behaviour Indicates Animal Personality in the Beadlet Anemone *Actinia equina* (Cnidaria)

**DOI:** 10.1371/journal.pone.0021963

**Published:** 2011-07-06

**Authors:** Mark Briffa, Julie Greenaway

**Affiliations:** Marine Biology and Ecology Research Centre, University of Plymouth, Drake Circus, Plymouth, United Kingdom; UC Santa Barbara, United States of America

## Abstract

‘Animal personality’ means that individuals differ from one another in either single behaviours or suites of related behaviours in a way that is consistent over time. It is usually assumed that such consistent individual differences in behaviour are driven by variation in how individuals respond to information about their environment, rather than by differences in external factors such as variation in microhabitat. Since behavioural variation is ubiquitous in nature we might expect ‘animal personality’ to be present in diverse taxa, including animals with relatively simple nervous systems. We investigated *in situ* startle responses in a sea anemone, *Actinia equina*, to determine whether personalities might be present in this example of an animal with a simple nervous system. We found very high levels of repeatability among individuals that were re-identified in the same locations over a three week sampling period. In a subset of the data, where we used tide-pool temperature measurements to control for a key element of variation in microhabitat, these high levels of repeatability remained. Although a range of other consistent differences in micro-habitat features could have contributed to consistent differences between the behaviour of individuals, these data suggest the presence of animal personality in *A. equina*. Rather than being restricted to certain groups, personality may be a general feature of animals and may be particularly pronounced in species with simple nervous systems.

## Introduction

Animal personalities are present when individuals show differences in behaviour that are consistent between situations, contexts or over time [1], [2]. Personalities have been demonstrated in both vertebrate (e.g. primates, birds and fish) and invertebrate (e.g. arthropods and molluscs) animals [3]. Understanding the taxonomic distribution of animal personality may shed light on its evolution. The key mechanism underlying personality is assumed to be variation in individual responses to environmental information such as risk levels [4], [5] or social context [6]. Therefore we should expect personality in any species where individuals might vary in how they gather, assess and respond to information about their environment. These processes do not require complex nervous systems, and in many organisms relatively simple systems for gathering and processing information are sufficient to enable appropriate responses to environmental cues [*7*]. In cnidarians, such as corals, jellyfish and sea anemones the nervous system consists of a non-centralised and diffuse ‘nerve-nets’ and the sensory cells are the simplest in structure of all metazoan animals [8].

In sea anemones (Cnidaria: Anthozoa), such as *Actinia equina*, a sedentary polyp is the dominant developmental phase and the medusa phase is absent. A polyp comprises a pedal disc attached to the substrate and linked by the column to an oral disc surrounded by feeding tentacles. *A. equina* is a solitary species and adjacent individuals frequently attack one another; the loser will slowly move away, leading to a well spaced distribution on rocky shores. Outside of aggressive interactions, however, movement may be infrequent [9]. They are highly tolerant to fluctuations in environmental variables such as emersion, temperature extremes and salinity extremes [9]. Although potentially long lived when maintained in aquaria, the typical life span of *A. equina* under natural conditions is approximately three years [9]. When disturbed, the anemone will retract its feeding tentacles to cover the oral disc, before re-opening. This ‘startle response’ is similar to that of withdrawing into a shelter when threatened, seen in species including poeceliid fish [10] and hermit crabs [11]. While the tentacles are held in this position the anemone is unable to feed or perform aggressive behaviour.

The aim of this study is to determine whether consistent between individual differences in the duration of startle responses are present in beadlet anemones, *A. equina*, under field conditions. Although highly tolerant of environmental fluctuations, we also aim to determine whether between-individual differences could be influenced by variation in microhabitat by investigating the effect of tide-pool temperature variation. If these simple animals have personalities we would expect significant repeatability in the duration of individual responses obtained on different occasions, irrespective of differences in microhabitat. A recent meta-analysis [12] indicates that, for many behaviours excluding courtship, arthropods show greater repeatability than vertebrate chordates. Therefore, providing further data on an additional phylum, the Cnidaria, will enhance our understanding of the taxonomic distribution of animal personality. Furthermore, most studies on behavioural repeatability are conducted under laboratory conditions (e.g. see [12]). Interestingly, an overall trend is for higher repeatability under field conditions compared to studies conducted in the lab [12]. Providing further data obtained during field studies is therefore necessary to increase our understanding of the causes of this difference. To facilitate cross-species comparisons with data obtained in other studies we provide three measures that have been used to quantify the ‘strength’ (i.e. the statistical effect sizes) of animal personalities in different studies.

## Materials and Methods

### Ethics statement

*Actinia equina* is not protected under either UK Law (Animals [Scientific Procedures] Act, 1986) nor listed in the general provisions (Article 1, Section 3) of the European Directive (2010) on the protection of animals used for scientific purposes. The University of Plymouth Ethics Committee has determined that specific ethical approval is not required for use of species that are not covered by the above provisions. However, the study was conducted in full accordance with the ASAB/ABS Guidelines for the treatment of animals in teaching and research. No animals were removed from their habitat or harmed in the process of conducting this study.

### Data collection

Data were collected *in situ* between September and December 2009 from two rocky shores, Mount Batten (UK grid reference: SX 48500 53117) and Wembury (UK grid reference: SX 51758 48377), located on the southwest coast of the U.K. Anemones were located attached to rocky substrates at both sites across the mid-shore, which was readily accessible at low tide. We selected anemones so that startle responses could be obtained at low tide, when tide-pools were accessible but anemones were still fully submerged in sea water with their feeding tentacles extended and oral discs exposed. Data had to be collected as the tide retreated but within tidal heights the order of data collection was varied between anemones. Thus, anemones that were located in groups of two to four tide-pools situated close together were startled in a different order on each occasion. Nevertheless, tide-pool exposure time could not be completely standardised in our sampling protocol (i.e. some anemones would have been in pools that had been exposed for longer than others at the time when data were collected) and we did not obtain data on how long each tide-pool had been exposed for prior to evoking the startle response. Therefore, the physicochemical properties of the sea water (e.g. temperature, oxygen content and pH) could have varied between tide pools as a result of different exposure times. Furthermore, the rate at which these variables changed following emersion would vary with factors such as the size of the tide pool and the amount of algal cover, and the height in the tide pool of the anenome. Therefore, for each anemone sampled at Wembury we also recorded water temperature at a distance of 1 cm from each anemone, using the probe of a digital thermometer, immediately following recovery from the startle response on all three occasions. Water temperature is not the only variable that might have differed between the microhabitats in which anemones were located but it is a key variable that influences metabolic rate in many marine organisms and co-varies with the oxygen content of water. Thus, variation in water temperature has been shown to be a key driver of differences in behaviour in other aquatic organisms [13]. The startle response was evoked by filling a 50 ml syringe with sea water from the rock pool containing the anemone and then rapidly discharging the syringe directly into the oral disc of the anemone from a distance of 2 cm. This caused anemones to retract their tentacles and the duration of the response was timed from the point at which the stimulus was applied to the point at which the anemone re-opened fully. The duration was recorded with a stopwatch and then converted into seconds prior to analysis. Care was taken to avoid direct contact between the syringe and any part of the anemone. Startle responses were obtained on three occasions: Occasion 1, then three days later (Occasion 2) and fourteen days later (Occasion 3). After the first startle response duration was recorded, an index of the size of each anemone was obtained by calculating the average of two measures of pedal disc diameter taken using digital callipers. In order to identify individual anemones on successive visits, a mark was made using nail varnish on the substrate near each individual. These marks were found to persist for the duration of the study. The distance and direction of each individual from the mark was also noted and a digital photograph of the anemone, the mark and the surrounding area of rocky substrate was taken as a further aid to re-identification. In five cases it was not possible to re-locate an anemone, possibly because they had moved, and the sample size was reduced from 70 to 65 (n = 29 Mountbatten, n = 36 Wembury). Data were only collected from anemones that showed no obvious signs of damage or disease and could be readily re-identified.

### Statistical methods

Initial analysis indicated that there was no difference in size between anemones from the two sites (unpaired t-test: t_63_ = 1.1, P = 0.3). A one-between, one-within repeated measures ANOVA indicated that startle response duration did not vary between occasions (repeated measure: *F*_2,126_ = 1.2, P = 0.3) or sites (the between-group factor: *F*_1,63_ = 0.006, P = 0.94) and there was no interaction effect *F*_2,126_ = 1.6, P = 0.22). Since neither morphological or behavioural variables differed between sites, the data from Mount Batten and Wembury were pooled in the following analyses, apart from those that incorporated temperature data, which was available for Wembrury only. A repeated measures ANOVA on the pooled data indicated that there was still no difference in mean startle responses between occasions (*F*_2,128_ = 0.95, P = 0.38) and a Levene's test for homogeneity of variance between sampling occasions in the pooled data indicated that there was no significant difference in variance between sampling occasions (*W* = 0.025, P = 0.98). Thus, any estimates of behavioural consistency were unlikely to be caused by unequal variances between time points, which can lead to spurious results [14].

To quantify individual consistency in the pooled data-set, repeatability (the intraclass correlation coefficient, ICC), *R_A_*, was calculated from the variance components obtained from a one-way ANOVA, testing for the presence of differences in mean responses between individuals (see [15] for the equation used to calculate *R_A_*, from the variance components shown in table 1). Repeatability may be calculated in a number of different ways [16], but in each case repeatability is a measure of the proportion of total variance accounted for by differences between groups (or ‘classes’) [16]. In the case of ANOVA based ICC this means the proportion of the total variance in a linear model that is accounted for by differences between individuals, where individual identities are treated as factorial predictors [16]. Repeatability in behaviour is therefore a function of both between-individual variation in responses and within individual (or ‘class’) correlation in responses. Repeatability will increase with the strength of both between individual variation and within-individual correlation in responses. Standard errors for *R*_A_ were calculated following [16]. Since many but not all studies use repeatability to assess individual consistency [12], we also included two further tests of individual consistency that have been used in previous studies; Kendall's coefficient of concordance [11] and, to provide a graphical illustration of any significant consistency (e.g. [11], [17]), the Pearson correlation coefficient. In order to include the full duration of the experiment the correlation between the durations of the first and third startle responses was calculated. Pearson's r was also calculated in order to test for the presence of a correlation between pedal disk diameter and startle response durations.

**Table 1:** Tests for difference in individual mean level startle responses and the variance components used to calculate repeatability for all data Repeatability±SE calculated from: *MS*_A_ = 96437.7, *MS*_W_ = 5865.0, *n*_0_ = 3, K = 65, N = 195.

	*SS*	*df*	*MS*	*F*	*P-value*
Individual	6172011.3	64	96437.7	16.4	<0.0001
Residual	762454.7	130	5865.0		
Total	6934465.9	194			

### Using ANCOVA to account for the effect of variation in temperature when calculating RA

For the sub-set of data from Wembury, for which temperature data were also available (i.e. not including the data from Mount Batten, for which we did not obtain temperature data), we calculated repeatability only. This is because we were able, in two distinct ways, to account for the effects of temperature on repeatability based on the methods of Nakagawa & Schielzeth [16] for dealing with confounds in the calculation of repeatability. We first calculated ‘*adjusted R_A_*’ by a method based on correcting for the effect of temperature, which is recommended for ANOVA based repeatability [16]. Nakagawa and Schielzeth [16] describe an example where the effects of a categorical cofound were adjusted for by applying a Z-transformation to the data [17]. The transformed data were then analysed using ANOVA and *R_A_* was calculated from the variance components as above. For continuous data such as temperature, an equivalent approach would be to de-trend the data, by obtaining residual startle responses from the relationship between temperature and startle responses, before performing the ANOVA. Alternatively, one could include the confound in the same analysis used to generate the variance components used to calculate *R_A_*, i.e. perform an ANCOVA, where the independent variable is ‘individual’, the dependent variable is ‘startle response duration’ and the co-variate is the temperature when each startle response was obtained. Thus, the variance components used to calculate *R_A_* have been derived from means that have first been adjusted for the effect of the co-variate [18] and represents a measure of how consistently individuals differ from the mean temperature-specific response.

While the above adjusted *R_A_* describes the repeatability once the effect of temperature has been removed (such that repeatabilities are calculated as if all measures were taken at the same temperature), a second way that the ANCOVA can be used to account for temperature is to retain its effect and calculate the amount of *R_A_* that remains when variation due to temperature is included but not controlled for. This gives a measure of the extent to which personality can ‘override’ the effect of a confound such as temperature, or to put it another way, how much personality ‘gets through’ the effect of temperature (or other conditional response). For ANOVA based repeatability, this can be calculated as in [15], with the following modification to the calculation of variance among individuals to include the effect of temperature: S^2^_A_ = (*MS*_A_−[*MS*_W_+*MS*_temp_])/*n*_o_. This is similar to the technique used to calculate ‘*conditionalR_A_*’ described by Nakagawa & Schielzeth [16] for non-Gaussian data.

A third advantage of factoring in temperature as a covariate is that we can compare the strength of the effects of ‘individual ID’ and ‘temperature’ on the mean duration of startle responses via comparison of the effect size estimate, partial Eta^2^ (*η* ^2^_p_), which represents the proportion of variance that is due to the effect of interest [11], [19].The *η* ^2^_p_ for ‘individual ID’ will increase with the magnitude of differences in means and decrease with variance around those means and will therefore behave in a similar way to *R_A_*. The *η* ^2^_p_ for ‘temperature’ will increase with the slope of the relationship between temperature and startle response duration and decrease with scatter around the slope. When the two effects are calculated from the same analysis, the values represent the proportion of variance that is driven by each effect.

For each test (repeatability, concordance and correlation for the pooled data, and repeatability only for the subset of data from Wembury), in addition to the effect size estimate commonly used to illustrate consistent between-individual differences (‘*R_A_*’, *η* ^2^_p_, ‘W’ and ‘r’) we also report the values of their respective test statistics (*F*, χ^2^ and *Z*) and their associated probability values.

## Results

**Figure 1:**
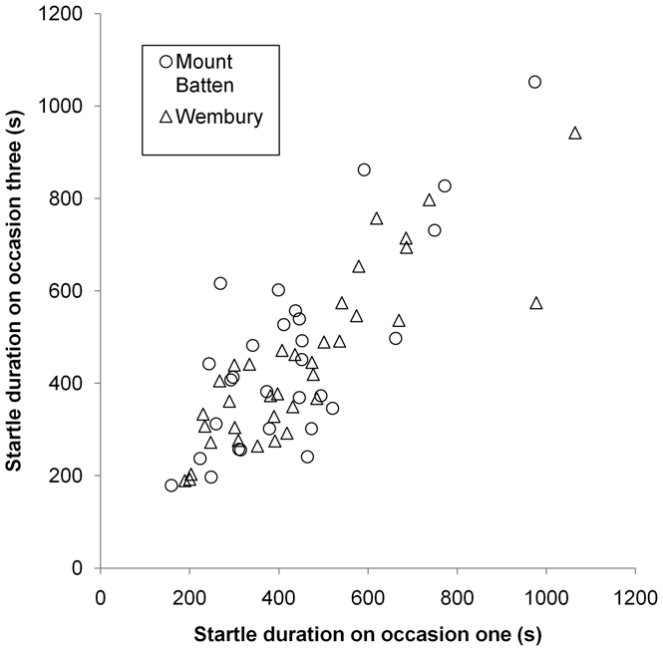
The strong positive correlation between individual startle response durations obtained on occasions one and three at the two study sites. Regression line added for illustration.

The ANOVA used to calculate *R_A_*±SE for the pooled data set is reported in table 1. There was significant repeatability (*R_A_* = 0.84±SE = 0.02; *F*_64,130_ = 16.4 P<0.0001) and concordance (W_64_ = 0.86; χ^2^ = 164.3, P<0.0001), in the duration of individual startle responses over three occasions and a significant correlation between occasions 1 and 3 (figure 1; r_63_ = 0.81; Z = 8.9, P<0.0001). Therefore there was a variation of 5% across the three estimates of behavioural consistency. These consistent differences in individual startle responses can be partially explained by variation in size as there was also a weak effect for mean startle response duration to increase with pedal disk diameter (r_63_ = 0.27; Z = 2.1, P = 0.033).

In the data from Wembury, the mean water temperature across all three occasions for all anemones was 10.38°C±SE = 0.04°C. Within anemones, the average temperature across the three occasions ranged from 9.73°C±SE = 0.26°C to 10.9°C±SE = 0.12°C. The ANCOVA used to calculate *R_A_*±SE for the sub-set of data from Wembury is reported in table 2. We first tested for homogeneity of slopes by examining the ‘temperature×individual’ interaction [18]. Since there was no significant difference between slopes (*F*_1,35_ = 1.2, P = 0.25) this effect was removed from the model [18]. Although there was a significant but weak negative effect of temperature on startle response duration (*F*_1,71_ = 4.1, P = 0.046, *η* ^2^_p_ = 0.05) there was still a strong pattern of repeatability in individual responses when temperature was adjusted for (*R_A_* = 0.90±SE = 0.001; *F*_35,71_ = 29.4 P<0.0001, *η* ^2^_p_ = 0.94). When the effect of temperature was retained in the calculation of *R_A_*, repeatability was reduced to *R_A_* = 0.62±SE = 0.001.

**Table 2:** Tests for difference in individual mean level startle responses and the variance components used to calculate repeatability for Wembury data.

	*SS*	*df*	*MS*	*F*	*P-value*
Individual	3620039.9	35	103429.7	29.4	<0.0001
Temperature	14532.7	1	14532.7	4.1	0.046
Residual	250058.6	71	3521.9		
Total	3884631.2	107			

Repeatability±SE calculated from: *MS*_A_ = 103429.7, *MS*_W_ = 3521.9, *MS*_TEMP_ = 14532.7, *n*_0_ = 3, K = 36, N = 108.

## Discussion

Animal personality has been documented in a wide range of study systems, including chordates (e.g. vertebrates) and in other phyla. A recent meta-analysis [12] indicates that repeatability is greater in field-based than in laboratory-based studies and, for behaviours excluding courtship, greater in invertebrates than in the vertebrates. The repeatability of *in situ* startle responses in *A. actinia* appears to be at the high extreme of the range seen in other field-based studies of both vertebrate and invertebrate animals. Here, we reported repeatabilities of 0.84 for the whole data set and an adjusted repeatability of 0.9 for the subset of data controlled for temperature, while repeatabilities reported in field based studies of other invertebrates included in the meta-analysis [12] ranged from 0.24 to 0.82 and for field based studies of vertebrates the range was 0.01 to 0.93. Relatively few personality studies have focussed on startle responses, where the animal is presented or manipulated with a threatening stimulus then the recovery time is quantified. In a study on the consistency of startle response durations in another intertidal invertebrate, the hermit crab *Pagurus bernhardus* (Arthropoda), which was conducted partially in the field [11], concordance was 0.55 compared to 0.86 for *A. actinia*. This shows the potential for marked differences in the repeatabilities of analogous behaviours between different invertebrate phyla, even when the studies were conducted under similar conditions and in a similar context.

Animal personality appears to be particularly strong in *A. equina*, but there is a key aspect of the biology of sea anemones that could account for some of the high repeatability reported here. While *A. equina* is not sessile, it is highly sedentary; indeed, we may have selected the least mobile individuals in our study, as only individuals that could be re-located in the same place between occasions were included in the analysis. Thus, over the course of the study period every individual remained in a specific location and due to the heterogeneous nature of rocky shores, they were likely to be subject to different microhabitats. One aspect of microhabitat that we controlled for was temperature, which had a weak but significant effect on startle response duration when included in the analysis of anemones from Wembury. It is likely that other components of microhabitat, such as position in the pool, exposure to tidal currents, exposure to wave action, prey availability and predation threat, which we did not quantify in this study, also varied between individual anemones, and could therefore also have contributed to consistent between-individual differences in startle response durations. Nevertheless, when *R_A_* is adjusted for temperature variation, such that its effect is statistically controlled for [16], there is still a high level of repeatability, indicating the presence of consistent between individual differences that are at least independent of temperature. Indeed, comparison of the partial Eta^2^ values shows that the effect of between-individual differences on startle response durations was far greater than the effect of temperature. On the other hand, when temperature is included in the calculation of *R_A_*, but not adjusted for, the repeatability is reduced to 0.62. This represents the personality effect that remains regardless of the effect of conditional responses due to temperature.

Although it has been recommended that confounds such as temperature variation, or other environmental factors that we did not measure here, are best avoided in studies of repeatability in behaviour [16], on some occasions the presence of such co-variates (or categorical factors in some cases) is unavoidable. Indeed, a greater understanding the role of environmental variables on repeatability may be desirable; given the apparent difference between field and laboratory repeatabilities that have been revealed by meta-analysis [12], it seems clear that more field based studies are required to complement those conducted in the laboratory. One approach for combining field and laboratory based studies might be to identify factors that influence repeatability in the field and then, using carefully designed experiments in the laboratory, to isolate their effects. Powerful factorial designs could be applied to investigate interactions between different factors that have been identified in the field. In this way factors such as temperature can be investigated experimentally rather than statistically. On the other hand, laboratory studies are unlikely to reflect the full complexity of conditions in the field and field-based studies of animal personality should not be neglected. Here we have demonstrated two ways of accounting for an uncontrolled environmental variable that allow us to (a) calculate repeatability as if all the behavioural measures were taken under the same conditions and (b) calculate the repeatability that remains when the effect of the variable is accounted for but not adjusted for.

In the case of sea anenomes and other sedentary or sessile animals, understanding the effects of such confounds is particularly important because animals that perform low rates of movement might be subject to consistent differences in microclimate that drive differences in behaviour. Similarly, hermit crabs, although highly mobile, occupy empty gastropod shells that act as ‘portable shelters’. Properties of these shells vary between individuals and variation in shell size [20] and colour morph [21] can influence the duration of consistent between-individual differences in startle response duration. However, as in the case of *A. actinia*, when these properties of microhabitat in hermit crabs are controlled for, consistent differences in behaviour that cannot be explained by differences in shell quality remain [20], [21]. Even in mobile animals that do not occupy portable shelters, such as fish [10], [22] or cephalopods [18] individuals might experience some level of consistent differences in habitat, which could contribute to consistent behavioural differences. For example, there might be habitat differences between territories. Thus, techniques for accounting for uncontrolled variables in the calculation of repeatability are likely to be useful for understanding the role of animal personality in natural settings in a range of study species.

‘Animal personality’ is considered to be present when groups of organisms show consistent between-individual variation in behaviour that is assumed to be a result of frequency dependent selection processes (e.g. natural selection; [5], sexual selection; [23]) and constraints on behavioural plasticity [24]. It is unclear whether similar mechanistic underpinnings contribute to animal personality across the diverse range of taxa in which it has now been documented, but typically ‘personality’ is thought of as denoting consistent between-individual differences in behaviour that are independent of obvious biological variables such as age or sex [2]. Thus, in most animals behavioural differences due to differences in microhabitat at the time of data collection might not be regarded as a component of personality. In sessile or highly sedentary animals, however, where consistent between-individual differences in mean microhabitat parameters can persist for significant proportions of life-spans, the case for excluding variation in microhabitat as a driver of animal personality is less clear.

In addition to variation in microhabitat, consistent between-individual differences may result from developmental changes such as learning [22] or life-history trade-offs [6], causing behavioural responses to vary with size. During an *in situ* examination of boldness in the poeciliid fish *Brachyraphis episcope*, for example, there was a strong positive correlation between size and the time taken to emerge from a shelter [25]. One explanation for such results is that between-individual differences in boldness are strongly influenced by size-dependent differences in metabolism [26]. While metabolic and activity rates are known to vary with body size in several animal phyla such as birds, mammals [27] and fish, less is known about these links in cnidarians, which lack discrete ventilation, circulatory and excretory systems [8]. Although ‘size-specific boldness’ was clearly present in the current data the effect size of this was weak. Therefore, regardless of how body size may be related to metabolism in *A. equina* body size alone cannot explain all of the between-individual variation in startle response duration.

In addition to factors such as metabolism and body size, it has also been suggested that personality may derive from constraints on behavioural plasticity [24], the ability of individuals to adapt their behaviour to new situations by responding to information about variation in their environment. Factors that will determine the expression of behavioural plasticity include the capacities for information gathering, assessment and decision-making. Although the ability of cnidarians such as *A. equina* to respond to changes in their environment has been documented, this has yet to be quantified in a way that would allow comparison with other study systems. Indeed, although there have been many studies of behavioural repeatability in invertebrates as a whole, at the taxonomic level the focus of work thus far is strongly skewed in favour of vertebrates. For example, Bell *et al.* [12] analysed 493 repeatabilities in chordates (all vertebrates) and 266 in arthropods (16 in arachnids, 4 in crustaceans and 246 in insects) but there were no examples of repeatability in other phyla. Although this large meta-analysis was not exhaustive of every study that has demonstrated the presence of animal personality (e.g. in molluscs, [28]), it seems clear that personality research currently focuses on a limited number of phyla with the majority of studies focussing on two subphyla, vertebrates and arthropods. More work on diverse invertebrate examples (perhaps including on other chordates) is needed in order to further elucidate the taxonomic distribution of animal personality. In the current study we show that different measures of behavioural consistency (*R_A_*, *W*, *r*) can yield similar results. While comparisons based on the same statistical measure, if available, may be preferable, comparing effect sizes between studies that have used these different statistical metrics may be a valid way to increase our understanding of personality in different taxa.

Although highly repeatable behaviour has been demonstrated in single celled prokaryotes [29], to our knowledge cnidarians are the metazoan taxon with the most simple nervous system tested in any personality study thus far. Previous studies have shown variation in aggressiveness between different genotypes of *Actinia tenebrosa* [30] but in the present study we have demonstrated, in the startle responses of *A. equina*, the first evidence of behavioural repeatability at the individual level in the Cnidaria. If constraints on plasticity do promote the existence of animal personalities then perhaps it is not surprising if they are particularly pronounced in animals such as *A. equina*, and other members of phyla that are characterised by simple body plans and nervous systems, which might allow for only limited behavioural plasticity.
